# Seeds and heavy metal: Defensin-like protein DEF8 mediates cadmium accumulation in rice and phloem unloading

**DOI:** 10.1093/plphys/kiac475

**Published:** 2022-10-12

**Authors:** Yana Kazachkova

**Affiliations:** Department of Molecular Biology, Princeton University, Princeton, New Jersey 08544, USA

Rice (*Oryza sativa*) is one of the most important staple crops worldwide, providing >20% of the calories to over 3.5 billion people (Wing et al., 2018). Cadmium (Cd) is a toxic heavy metal that affects the growth and metabolism of plants. Exposure to Cd is also linked to potential health risks in humans (Hayat et al., 2019). Most nonwork-related Cd exposure is associated with food, with rice being a major source of human Cd intake. Hence, understanding Cd accumulation mechanisms in rice could enable controlling Cd content in rice grains (Clemens and Ma, 2016).

Cd uptake by roots and its translocation within a plant are tightly regulated by transporter activity. To date, several transporter proteins associated with Cd uptake in roots have been identified, including Natural Resistance-Associated Macrophage Protein 5 and 1 (OsNRAMP5/1) and the major facilitator family protein OsCd1 (Sasaki et al., 2012; Yan et al., 2019; Chang et al., 2020). Another Cd transporter, OsHMA3 (Heavy Metal ATPase 3), localizes to the tonoplast membrane of rice root cells and participates in Cd sequestration into root vacuoles, thereby preventing Cd toxicity in aerial plant parts (Ueno et al., 2010). OsLCT1 (Low-affinity Cation Transporter 1), a low-affinity cation transporter, mediates Cd loading into the phloem, regulating Cd transport into rice grains (Uraguchi et al., 2011). However, no phloem unloading transporters have been identified to date.

In this issue of the *Plant Physiology*, **Tian-Yu Gu and colleagues** (Gu et al. 2022) demonstrate the role of defensin family member DEF8 as a mediator of xylem Cd loading and phloem Cd unloading in rice. Defensins are a group of cysteine-rich proteins composed of the cysteine-rich domain that can bind to metal ions and the secretion signal peptide (SSP). Several defensin-like proteins play a role in Cd transport and distribution, suggesting that other defensin family members could play important roles in Cd translocation within a plant (Luo et al., 2018).

First, the authors studied gene expression profiles of all rice defensin family genes. *DEFENSIN 8* (*DEF8)* was selected for further characterization due to its high expression levels in rice endosperm. *DEF8* expression was upregulated in seeds through maturation and in roots during Cd exposure, suggesting that *DEF8* participates in rice grain Cd loading.

**Figure 1:**
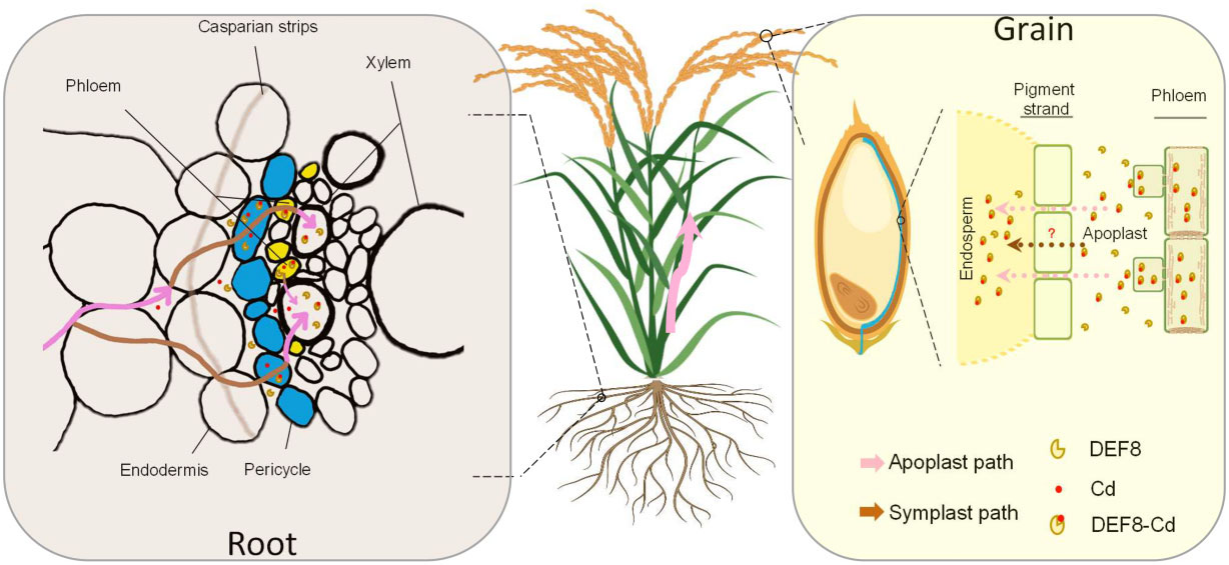
Schematic model of Cd allocation in rice mediated by DEF8. In roots, *DEF8* is expressed in pericycle and phloem cells. DEF8 enables Cd efflux to the apoplast and xylem for long-distance transport to aerial parts of the plant. In the grain, Cd from the apoplast accumulates in the endosperm either by diffusion or active transport mediated by unknown transporter(s). DEF8–Cd indicates a hypothetical complex of Cd and DEF8. The figure is based on Figure 8 from Gu et al. (2022).

Histochemical analysis showed that *DEF8* was expressed in the roots and shoots of rice seedlings, specifically in phloem and pericycle cells (Figure 1). Fluorescently tagged DEF8 localized to cell walls of rice sheath and onion (*Allium cepa*) epidermis cells. Interestingly, when the SSP domain sequence was removed, DEF8 re-localized to the cytosol, indicating that the SSP domain is indispensable for DEF8 efflux. Taken together, DEF8 tissue and subcellular localization indicated its possible role in Cd long-distance transport.

Plant treatment with BrefeldinA, a vesicular transport inhibitor, led to the formation of vesicle bodies in *Arabidopsis thaliana* roots, suggesting that DEF8 could be secreted through the vesicle trafficking pathway. Binding assays with recombinant full-length and mature (lacking SSP domain) DEF8 at acidic and neutral pH showed Cd chelation activity, indicating that DEF8 could mediate Cd transport to the apoplast via chelation and secretion.

To determine the role of DEF8 in rice plants, the authors created *DEF8* overexpression and knockout lines. The mutant lines and respective wild-type controls were hydroponically grown in media supplemented with Cd. Plants overexpressing *DEF8* had lower levels of Cd in roots and higher levels in aerial parts of the plant, xylem, and guttation fluid, while the knockout plants exhibited an opposite trend. Overexpression of *DEF8* in Arabidopsis led to lesser inhibition of root growth and lower Cd root levels when grown on Cd-supplemented media. These results suggest that in rice seedlings DEF8 mediates Cd long-distance transport from roots to shoots via Cd xylem loading. These findings also suggest a possibility to employ *DEF8* expression to modulate Cd accumulation in other plant species.

To investigate the role of DEF8 in Cd accumulation in rice grains, the authors carried out detailed grain histochemical analysis and showed that *DEF8* was expressed in vascular bundles of glumes at the seed heading stage, while at the grain filling stage the expression was only observed at the vascular bundle of the endosperm. Cd accumulation decreased in brown rice and the rachis of the *def8* mutant, whereas in both overexpression lines, more Cd was observed in brown rice but not in the rachis. These results suggest that DEF8 mediates Cd unloading from phloem during rice grain filling (Figure 1). Since no differences were observed in accumulation of other metal ions, the findings indicate that DEF8 has a specific role in Cd translocation via phloem or xylem, depending on the plant developmental stage. It will be interesting to further explore the mechanism of Cd transport by DEF8 and the role of vesicular structures that the authors observed after BrefeldinA treatment. Considering that most Cd in soils is of anthropogenic origin, the question about other roles of DEF8 in the absence of Cd remains open.

It is interesting that, in contrast to other transporters, DEF8 exhibited specific transport activity for Cd but not for other metals, suggesting that genetic manipulations with *DEF8* will not affect the homeostasis of other metal ions in the plant. When grown on Cd-polluted soil, no significant yield or phenotypic changes were observed between wild-type, knockout, and overexpression plants. Taken together, these data make *DEF8* a desirable target for genetic manipulations to create low-Cd rice and potentially tailor Cd accumulation in other plant species.

*Conflict of interest statement*. None declared.
